# The Monogenean Parasite Fauna of Cichlids: A Potential Tool for Host Biogeography

**DOI:** 10.4061/2011/471480

**Published:** 2011-08-13

**Authors:** Antoine Pariselle, Walter A. Boeger, Jos Snoeks, Charles F. Bilong Bilong, Serge Morand, Maarten P. M. Vanhove

**Affiliations:** ^1^ISE-M, UMR5554 CNRS, UR226 IRD (ex-ORSTOM), Université Montpellier II—CC 063, 34095 Montpellier Cedex 5, France; ^2^Laboratório de Ecologia Molecular e Parasitologia Evolutiva, Grupo Integrado de Aquicultura e Estudos Ambientais, Universidade Federal do Paraná, Setor de Ciências Biológicas, Departamento de Zoologia, Caixa Postal 19073, CEP 81531-980, Curitiba, PR, Brazil; ^3^Ichthyology Unit, African Zoology Department, Royal Museum for Central Africa, Leuvensesteenweg 13, 3080 Tervuren, Belgium; ^4^Laboratory of Animal Diversity and Systematics, Biology Department, Katholieke Universiteit Leuven, Charles Deberiotstraat 32, 3000 Leuven, Belgium; ^5^Laboratoire de Parasitologie et d'Ecologie, Département de Biologie et Physiologie Animales, Université de Yaoundé I, BP 812, Yaoundé, Cameroon

## Abstract

We discuss geographical distribution and phylogeny of Dactylogyridea (Monogenea) parasitizing Cichlidae to elucidate their hosts' history. Although mesoparasitic Monogenea (*Enterogyrus* spp.) show typical vicariant distribution, ectoparasitic representatives from different continents are not considered sister taxa, hence their distribution cannot result from vicariance alone. Because of the close host-parasite relationship, this might indicate that present-day cichlid distribution may also reflect dispersal through coastal or brackish waters. Loss of ectoparasites during transoceanic migration, followed by lateral transfer from other fish families might explain extant host-parasite associations. Because of its mesoparasitic nature, hence not subject to salinity variations of the host's environment, *Enterogyrus* could have survived marine migrations, intolerable for ectoparasites. Host-switches and salinity transitions may be invoked to explain the pattern revealed by a preliminary morphological phylogeny of monogenean genera from Cichlidae and other selected Monogenea genera, rendering the parasite distribution explicable under both vicariance and dispersal. Testable hypotheses are put forward in this parasitological approach to cichlid biogeography. Along with more comprehensive in-depth morphological phylogeny, comparison with molecular data, clarifying dactylogyridean evolution on different continents and from various fish families, and providing temporal information on host-parasite history, are needed to discriminate between the possible scenarios.

## 1. Introduction: Explanations to the Current Distribution Pattern of Freshwater Fish Groups

Organisms with limited dispersal abilities are generally considered to be useful tools in historical biogeography. Examples include amphibians [1] and freshwater fishes [2, 3]. At the heart of many discussions on the evolutionary history and distribution patterns of major freshwater fish groups is the vicariance *versus* dispersal debate (e.g., [4, 5]). Although vicariance-based scenarios have classically been favoured, de Queiroz [6] gives an overview of how the importance of (often seemingly unlikely) dispersal events has been underestimated in historical biogeography, though his examples stem mostly from plants and terrestrial biota. 

It is generally accepted that the distribution of several ancient freshwater groups such as Dipnoi (lungfishes) and Osteoglossiformes (bony tongues) results from major vicariant events after the breakup of Gondwana [7]. However, because of conflicting evidence, the discussion continues for more recent groups. Hertwig [8] discussed the biogeographic implications of the phylogeny of the Cyprinodontiformes (rivulines, killifishes, and live bearers). He explained their distribution by vicariance events in the basal clades, combined with subsequent dispersal, and stressed the discrepancy of the vicariance hypothesis with the paleontological record. For example, in aplocheiloid killifishes, the fossil record is much younger than the supposedly African-South American drift-vicariance event [9]. 

The same is true within the Characiformes (characins), for example, in Alestidae; Zanata and Vari [10] forwarded a clear vicariance hypothesis to explain their distribution and relationships. In addition, they mention typically vicariant biogeographic patterns in two other groups of Characiformes with African-South American sistergroup relationships. However, Calcagnotto et al. [11], in a molecular analysis, mostly confirming earlier morphology-based trees, concluded that marine dispersal cannot be excluded *a priori* and that a simple model of vicariance could not explain the biogeographic history of the order. 

Within the Siluriformes (catfishes), the situation is more complex since several taxa are marine inhabitants. Sullivan et al. [12] could not confirm the existence of a supposedly trans-Atlantic clade suggested by others, but conversely, their overall tree of Siluriformes did not contradict a general vicariant distribution pattern of tropical freshwater catfishes either. A few recent publications have offered new angles to the vicariance *versus* dispersal debate. To explain the unexpected discovery of a Mesoamerican catfish within an African clade, Lundberg et al. [13] postulated a northern ancient intercontinental passage in warmer periods, including dispersal through freshened warm surface waters of the Arctic and adjacent oceans. Such episodic fresh surface waters have been suggested by Brinkhuis et al. [14] as an explanation for the presence of the freshwater fern *Azolla *and freshwater microfossils in Eocene marine deposits.

## 2. The Case of Cichlidae

For Cichlidae, the discussion is far from over either, as they exhibit a comparable biogeographic pattern found in Cyprinodontiformes, with basal lineages occurring in India and Madagascar, and the common problem of discordance between their fossil record and their age under the vicariance hypothesis [8]. Most recent arguments, however, seem to favour the vicariance model (e.g., [15–17]), but the dispersal model could not be eliminated. In fact, the numerous studies that have examined the fossil record and morphological and/or molecular phylogenies of the world's cichlid fauna (e.g., [9, 15, 17–29]) have not been able to end all doubts on the choice between the two main hypotheses explaining their current distribution pattern. 

The first hypothesis postulates that the cichlid fishes originated ca. 130 million years ago (MYA) in Gondwana. Their current disjunctive distribution area (Africa, including Madagascar; South and Central America, Texas and the Caribbean; southern mainland India and Sri Lanka; the Levant and Iran [30]) comprises mostly Gondwanan regions; hence cichlids would have already been present in the major part of their current range before the splitting up of this super-continent. As for their non-Gondwanan occurrence in Texas, the Caribbean, the Levant, and Iran, migration through river systems over more recent landbridges is assumed (e.g., [16]) though the dispersal mode is still debated for Central America and the Caribbean [31, 32]. This implies that cichlid evolutionary history and present large-scale distribution on the continents resulted from major vicariant events and that cichlids did not need to cross extensive marine barriers to reach these distribution areas. 

The second hypothesis, in line with the fossil data, suggests that cichlid fishes originated near Madagascar, or more precisely, that the cichlids living there belong to ancestral lineages, in view of their paraphyly ([25, 33, 34] and references therein). Consequently, cichlids would have secondarily colonized their current disjunctive distribution areas [24–26]. This scenario assumes that cichlid evolutionary history and present distribution have resulted from dispersal across various marine water channels. 

According to Chakrabarty [15], the only way to resolve the controversy of the origin of Cichlidae (except for the discovery of a cichlid fossil older than 65 MY) is to falsify either of the two hypotheses. As the dispersal hypothesis is untestable because any distribution pattern can be explained by dispersal, Chakrabarty [15] suggested four potential means to show the vicariance hypothesis incorrect. Three of these falsifiers are based on the demonstration of an incompatibility between the timing of two supposedly concomitant events (one linked to the divergence of lineages, the other to geological processes); the fourth one includes the discovery of the same cichlid species on both sides of a supposed barrier to dispersal. 

One of the potential “timing” falsifiers involves the use of molecular clock estimations, the absolute accuracy of which still remains unknown (e.g., [35] *versus* [36, 37]). However, Azuma et al. [17] made a strong case in calculating divergence times of major cichlid lineages, based on molecular evolutionary rates of large mitogenomic datasets of six cichlid species, leading to additional support for the vicariance hypothesis. As for the discovery of a new cichlid species on both sides of a marine channel, the likelihood is very low. The same applies to the finding of a cretaceous cichlid fossil. In comparison with other freshwater fishes spanning such a period of time, this would indeed imply a relatively large gap in the fossil record [25]. Furthermore, the advanced position of perciformes in teleost phylogeny (several higher level taxa encompassing the cichlids do not appear before the Gondwanan breakup) and the extensive fossil record available of several cichlid lineages make such a discovery improbable [38].

## 3. Parasites as an Additional Source of Information

Another solution might come from the use of a separate and independent data set related to cichlids. It is well established that parasites can furnish information on their hosts' ecology and (past and current) distribution [39–42]. Following Hoberg [43] and Nieberding and Olivieri [42], we could use parasites as keystones in biogeography, or proxies, to provide a new dimension to understand ecological interactions, distribution patterns, and the history of geographic regions and biota. 

Some work has been done in this framework to infer host biogeography [44–55]. More specifically, interesting examples include the reconstruction of biogeographical events through the analysis of parasite communities [56–59] and the detection of patterns at a higher resolution than host genetics would permit [60–62]. Parasite phylogenetic data can also yield supporting or complementary information on host phylogenies [63–67]. Surprisingly, few studies refer to fishes and their associated monogenean parasites whereas these organisms seem useful as indicators of host biogeography and phylogeny. Indeed, they are strictly parasitic (i.e., adults cannot survive for a long time as free-living organisms), holoxenous (i.e., they have a direct lifecycle, with a short free-living larval stage which actively infests a new host), and generally oioxenous (i.e., parasite species are often species specific with regard to their host). A limited number of cases were made using these parasites, applying them in the fields of genetic population substructuring [68], historical patterns of their hosts' dispersal [69] or distribution [70], (co-)phylogenetic patterns [71–73], and host identification [74]. 

Pariselle et al. [75] used data from West African cichlids and concluded that monogenean species can behave similarly to the alleles of genes of their fish hosts. Distribution, and therefore biogeography, of the hosts will directly affect that of their monogeneans. Conversely, information provided by these parasites should be very useful for studies in fish biogeography. Importantly, as demonstrated by Pérez-Ponce de Léon and Choudhury [59] for South and Central American cichlids, a phylogenetic study of both host and parasite taxa is paramount to infer hypotheses on historical biogeography. Evidently, a geographically restricted availability of parasite records can seriously hamper an analysis. For example, theories on the “original” host, needed to infer host-switching or dispersal pathways, are hard to infer when certain regions or host taxa are undersampled. In addition, insufficient sampling ensues obvious problems related to how “real” absence data are when considering community composition [58].

Here we illustrate how parasite information may complement the discussion on vicariance *versus* dispersal hypotheses for cichlid age and distribution processes.

## 4. The Monogenean Parasite Fauna of Cichlids

### 4.1. Data Collection on Cichlid Parasites

Because of the oioxenous host specificity of many monogenean species, the (geological) time scale and the biogeographical distribution of their hosts (no genus is represented naturally on two different continents) [76, 77], the approach used within the framework of this study is based on the generic rather than the species level. Some ancyrocephalid monogeneans from South American and African cichlids are known to exhibit a relatively low host specificity, infesting several host species [78, 79]. However, their otherwise often narrow species specificity would, in our view, render examining the parasite data at species level equivalent to the use of autapomorphic characters (instead of synapomorphic ones) in a phylogenetic tree, which is not appropriate [73] to infer the biogeographical history of the host. Furthermore, there are considerable differences in the number of parasite species reported from the various host genera. In view of the relatively low proportion of cichlid species worldwide examined for the presence of Monogenea, this imbalance is more likely to reflect differential sampling effort than variations in species richness between genera. Conversely, on the genus level, we feel confident the current state-of-the-art approaches the actual parasite diversity on cichlids to a higher extent. 

**Figure 1:**
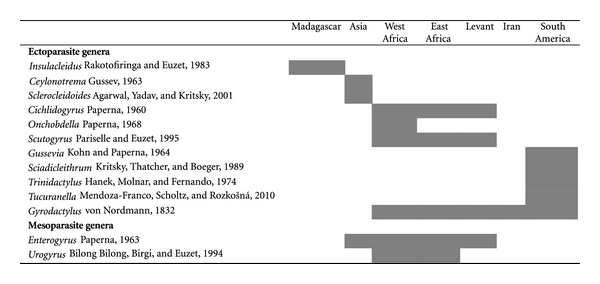
Current state of knowledge on biogeographical distribution of monogenean genera from cichlid fishes (fields shaded in grey indicate presence), compiled on the basis of [78, 82–90] and additional unpublished data from the authors. Note that sufficient data are clearly lacking for some regions, for example, on whether or not Malagasy and South American cichlids host mesoparasitic Monogenea. Recent synonymisations are taken into account, as are *nomina inquirenda*, for instance *Oreochromogyrus* Ferdousi and Chandra, 2002, which most likely concerns misidentified *Cichlidogyrus* larvae [78]. Only records from hosts occurring under natural conditions on the respective continents are included.

To the best of our knowledge, Figure 1 provides a complete overview of the 13 monogenean genera infecting cichlids. Unfortunately, a considerable proportion of available data is fragmentary and based on studies on a limited number of regions or host species. The most exhaustively studied assemblage is probably Ancyrocephalidae from West African tilapias. The exemplary nature of monogenean records worldwide is demonstrated by the fact that, while there are revisions and phylogenies available for certain well-defined taxa (e.g., Diplectanidae [80], Capsalidae [81]), comprehensive worldwide information on Monogenea of an entire fish family is extremely rare.

The worldwide distribution of monogenean genera described from cichlid hosts shows a clear difference between the distributions of ecto- (i.e., living on the host, directly in contact with the environment) and mesoparasite (i.e., living in a host body cavity and not in direct contact with the external environment [91]) genera. The mesoparasite *Enterogyrus *is present in Asia, the whole of Africa, and the Levant. Pariselle and Euzet [78] hypothesize that the Asian and African-Levantine representatives of this genus might be considered as belonging to separate genera, on the basis of the possession of two *versus* one haptoral transversal bar. However, the overall similarities in haptor and even male copulatory organ structure suggest the various *Enterogyrus *spp. to be very closely related anyway. 

In contrast, all ectoparasitic genera are endemic to the continent where they occur. Two genera currently seem to display a more restricted distribution: *Onchobdella* and *Urogyrus*. The first one was expected, like other African ectoparasitic genera, to be present on the entire continent, and not only in West Africa, but was found to be specific to hosts whose distribution is limited to this region (*Hemichromis* Peters, 1858 and *Pelmatochromis* Steindachner, 1894). *Urogyrus*, being a mesoparasite like *Enterogyrus*, could potentially be present on several continents, but is only found on hosts whose distribution is restricted to Africa (including haplochromines from the East African Great Lakes [92]). 

While the above-mentioned cichlid parasites are mostly dactylogyridean Monogenea, the nominal (albeit probably paraphyletic [93]) genus *Gyrodactylus *is a member of the Gyrodactylidae, first proposed by Van Beneden and Hesse [94]. Moreover, despite its extensive morphological plasticity (*cfr. infra*), *Gyrodactylus *species are rather conservative on the scale of the characters used in this study. Given the limited number of representatives known from cichlids, this genus would not yield a high resolution in a morphological phylogeny. As we need a cladistic analysis of the genera under study, *Gyrodactylus *will not be included in our investigation. In contrast, it should be noted that *Gyrodactylus* in itself could be a useful biogeographic tool [95], as can other gyrodactylids [70]. However, the limited molecular data available on African and South American *Gyrodactylus *species do not show any close affinities between them either [82].

### 4.2. Phylogenetic Analysis

A preliminary phylogenetic analysis was performed using selected morphological characters available in the literature. Genera included are all those known to parasitize cichlids and selected parasites from marine and freshwater perciform fishes (Table 1) from the different continents home to cichlids. *Quadriacanthus*, infesting siluriformes, was added as its representatives depict similar and comparable morphological features (e.g., hook morphology). The inclusion of these taxa allowed a preliminary test of the monophyly of the parasites of Cichlidae, which would be the expected pattern under cospeciation (either by vicariance or dispersal). The addition of genera from other fish groups provided a preliminary test that the parasitic fauna of cichlids could encompass sister lineages to parasites of other sympatric fish species, especially if the hypothesis of parasite loss during dispersal in marine waters was supported.

The hypothesis on their relationship was proposed based on parsimony analysis of 17 unordered homologous series (Appendix). The chosen putative homologous series are those considered less prone to errors introduced by incomplete or questionable interpretations of morphology in the original descriptions of the species. Five of the homologous series used pertain to the copulatory complex; all others, to the haptoral elements. Although both haptoral and copulatory complex, next to soft body parts, are important in Monogenea systematics [103], many other scientists focus their analyses solely on the attachment organ (e.g., [104–106]). Its complexity and variability, as well as the higher number of comparable elements, make it easier to extract coded characters from the haptor than from the genitals. Moreover, several studies comparing morphological and molecular data in Ancyrocephalidae suggest that the attachment organ structure mirrors phylogenetic relationships on the level between genera or major lineages while the copulatory organ is more suitable for distinguishing closely related species [107, 108]. The phylogenetic hypothesis was constructed in PAUP* v.4.0 b10 [109], with the Bremer support index [110–112] calculated with the help of TreeRot v.3.0 [113]. Initial analyses, using heuristic search (under a tree-bisection and reconnection branch swapping algorithm with 1000 random-addition-sequence replicates) provided individual consistency indices for character states used in successive weighting procedures [114] until values of the overall consistency (CI: [115]) and retention indices (RI: [116]) stabilized. 

**Figure 2:**
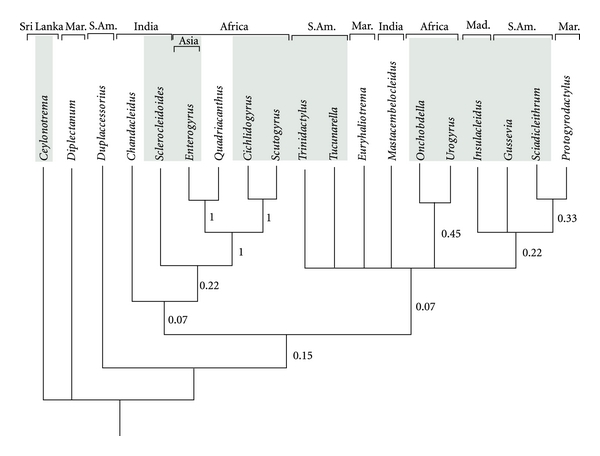
Strict consensus cladogram (of 21 EPT) depicting the putative phylogenetic relationship of Monogenea from Cichlidae (shaded rectangles) and other host groups. As branch support, Bremer support values are shown. Geographic distribution of species of each genus is depicted above generic names (“Mar.”: marine; “Mad.”: Madagascar; “S. Am.”: South America).

A total of 21 equally parsimonious trees (EPT) resulted from the analysis of parsimony (length = 13.97; CI = 0.81; CI excluding uninformative characters = 0.76; RI = 0.85). The strict consensus cladogram, summarising the most parsimonious phylogenetic relationships recovered from all EPT between the analysed genera is presented in Figure 2. The genera with species limited to Cichlidae (shaded genera names on Figure 2) are represented by up to three independent groups. The phylogenetic hypothesis does not support a single monophyletic assemblage of parasites of Cichlidae.

## 5. Information to Be Drawn from Cichlid Monogeneans

Our review shows that dactylogyridean ecto- and mesoparasites of cichlid hosts differ in their distributions. This raises the question as to which of the above-mentioned hypotheses on the biogeographic pattern of cichlids best explains this distributional incongruence. We would expect similar patterns in their geographic distribution, assuming that all lineages, meso- and ectoparasitic, have an equally long association with their cichlid hosts, a similar evolutionary rate, and have equally been subjected to the same biogeographical and coevolutionary processes/events. The African ectoparasitic *Cichlidogyrus* (as well as the mesoparasitic *Enterogyrus*) was found to infect South American cichlids following introduction of their African hosts, and the American *Sciadicleithrum* was reported from African cichlids under artificial conditions [117]. This supports the view that the evolutionary divergence between African and Neotropical cichlids is not the reason they do not share ectoparasites. 

**Figure 3:**
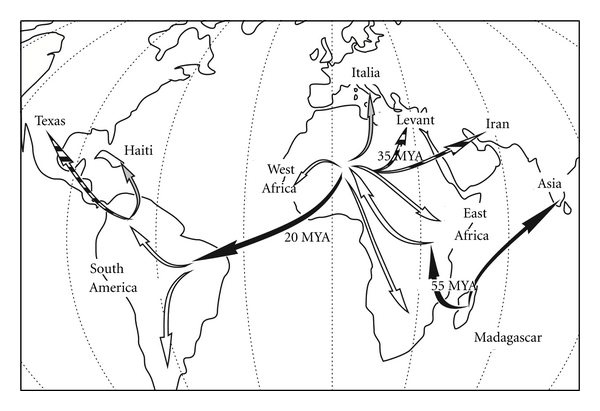
Simplification of Murray's hypothesis [25] on the origin and biogeography of the world's cichlids. Arrows symbolising dispersal routes are indicated as follows: marine (black); freshwater (white); unresolved (black and white); unknown (fossils only; grey).

Thus, it is likely that the source of discrepancies between the distribution patterns observed for meso- and ectoparasites resides in the fact that environmental factors may influence these parasite communities differently (e.g., [118]). In the framework of the dispersal hypothesis (see [25]), there is a single such factor that may influence the two types of parasites in two different ways. This theory assumes that there were successive migrations, taking place in different environmental conditions. Some hosts are assumed to have crossed marine waters (between Madagascar and Asia; Madagascar and Africa; West Africa and South America; Figure 3, black arrows) while others only used freshwater dispersal pathways (dispersal within Africa or South America; Figure 3, white arrows). Ectoparasites are directly affected by changes in the environment while mesoparasites such as *Enterogyrus* found in the stomach are not. Does this differential exposure to saline water allow us to favour one of both hypotheses on cichlid history? A couple of issues need to be addressed before this question can be answered.

### 5.1. Salinity Tolerance of Cichlidae and Monogenea

Many cichlids exhibit tolerance to a broad salinity range and some even display a mostly brackish lifestyle, such as representatives of the Asian *Etroplus *Cuvier, 1830, the only Iranian endemic cichlid *Iranocichla hormuzensis* Coad, 1982, and some African, Malagasy, and Neotropical species ([25] and references therein, [30, 32, 119–129]). Murray [25] and Briggs [9] used this ability as an important argument in favour of the recent dispersal hypothesis, but Sparks and Smith [16] contradicted the long-time survival of any cichlid species in saltwater conditions. Although, at least under natural conditions, there are no fully marine cichlid species at present, several wild populations occur in (highly) saline environments (e.g., *Sarotherodon melanotheron *Rüppell, 1852 in the Gambia and Senegal rivers [130], in Hann bay (off Dakar), Senegal and in Saint Jean bay, Mauritania; *Tilapia guineensis* (Günther, 1862) in Hann bay (off Dakar), Senegal (A. Pariselle, pers. obs.)).

Cichlids were possibly able to survive in marine environments, but this may not have been the case for their monogenean parasites, whose tolerance to salinity variations is generally shown to be low. Indeed, osmotic shocks are commonly used as a treatment against ectoparasitic Monogenea [123, 131–134]. Pariselle and Diamanka [135] showed that *S. melanotheron* lost all monogenean gill parasites with the increase of water salinity (>35 g/L), both in a natural environment and under experimental conditions. 

On the other hand, *Enterogyrus* has been reported from highly saline waters [83]. The biogeography of mesoparasites (*Enterogyrus*) seems to display a vicariant history, as the same genus is present in Asia and Africa (Figure 1). The lack of shared ectoparasites between continents and the most often limited (as compared to their hosts') salinity tolerance of *Cichlidogyrus* is mentioned by Paperna [83] as an argument for the marine dispersal theory. Indeed, one could hypothesize that marine dispersal events in cichlids caused them to lose their ectoparasites, while retaining their mesoparasites, unexposed to the saline water. This could explain the incongruence between the distribution patterns of ecto- and mesoparasitic Dactylogyridea. However, for this conclusion to be drawn, it is necessary to know the interrelationships between the various monogenean genera under study.

### 5.2. Lessons from the Cladistic Analysis

The morphology of Monogenea and other flatworms is prone to display homoplasy, suggesting molecular phylogenetics to be needed to conclusively resolve their evolutionary relationships [81, 136, 137]. Indeed, plasticity has been shown both in haptoral [138–142] and genital [143–145] structures. This demonstrates that not only phylogeny but also geography, host-related and environmental factors, and (sexual) selection may influence the morphology of monogenean hard parts. On the other hand, most of this variability is continuous/quantitative and between conspecific individuals, and, hence, should not represent a significant interference in construction of phylogenetic hypotheses based on discrete characters differing between genera. Moreover, ambiguities or resolution problems related to morphological phylogenies were mostly shown in groups of organisms displaying taxonomic difficulties and a limited number of discretely varying haptoral characters (e.g., gyrodactylid Monogenea [137]), lacking sufficient characters comparable throughout the family (e.g., six in capsalid Monogenea [81]), or where plastic characters, prone to loss or acquisition, were used (e.g., mouthpart structure reflecting trophic adaptation in lysianassoid Amphipoda [146]). In contrast, based on a limited number of species of ancyrocephalid Dactylogyridea, there are clear indications that haptor morphology contains phylogenetic signal [107, 108]. On the level within or between genera, haptoral (and, on the between-species level: genital) structure does not conflict with molecular taxon boundaries and is systematically informative. Indeed, on these levels, geography or host characteristics do not seem to influence haptor morphology in this monogenean family. 

The only genetic data on monogeneans infecting cichlid fishes stem from African species [100, 107], and are therefore uninformative for intercontinental comparison. Hence, a morphological phylogenetic hypothesis, based on a combination of haptoral and genital elements, seems a reasonable approach given the currently available knowledge. As any scientific hypothesis, it is prone to extensive reconsideration once sufficient genetic data are collected, as such data will yield a higher number of informative characters [147] compared to the presently available morphological knowledge. The morphological phylogenetic hypothesis (Figure 2) is fundamentally compatible with the results of the molecular phylogeny proposed by Mendlová et al. [100], with some more conspicuous coincidences (e.g., on the sistergroup relation of *Scutogyrus* and *Cichlidogyrus*). Also the close affinity between *Ceylonotrema *and *Diplectanum* might merit further scrutiny, as the ventral and dorsal bars of *Ceylonotrema *indeed show similarities to those in diplectanids. As molecular phylogenetics also faces, among others, problems with homoplasy [148–151], combining insights from both phenotypic and genetic classification in testing hypotheses and identifying uncertainties seems the most fruitful way forward (e.g., Rota-Stabelli et al. [152] for deep phylogeny of arthropods).

Although the above-mentioned flaws limit the interpretation we can give to our tree reconstruction in Figure 2, which should not be regarded as a definitive hypothesis on dactylogyridean evolutionary history, this tree provides the first comprehensive interpretation on phylogenetic relationships among cichlid monogeneans. Although based on morphological characters, it represents a phylogenetic hypothesis that may be tested based on molecular data. Furthermore, the CIs and RI of the tree demonstrate the existence of phylogenetic signal, independent of the number of homologous series available.

The cladogram indicates that parasitizing on cichlids is a polyphyletic character in Dactylogyridea. The non-sistergroup relationship of the ectoparasitic Dactylogyridea of cichlids on the different continents seems an interesting argument to support the dispersal hypothesis. On the other hand, if the parasites distribution is to be explained mainly by oceanic dispersal (of their hosts), one would expect their phylogenetic relationships to follow continental borders (as suggested for Gyrodactylidae by Boeger et al. [95]). The ectoparasite genera of different continents, however, are not each other's sister taxa based on the morphological cladogram (Figure 2). Hence, host-switching events from other fish groups are required, under whatever scenario, to explain the host-parasite distribution observed in the preliminary cladogram. Therefore, the evolutionary history of other fish families serving as “source hosts” should be considered. 

Because Monogenea with a marine lifestyle appear as sister taxa to several cichlid (and thus, freshwater) parasites in our cladogram, lateral transfer from other (including marine, necessitating freshwater/marine transition) hosts could be needed to explain our cladogram, irrespective of the cichlid scenario (Figure 2). Host-switching [153], even between fish hosts that differ at the ordinal level [154], as well as substantial salinity tolerance within species [155] have been observed in Monogenea. Although both have mainly been demonstrated in gyrodactylids, even representatives of the ancyrocephalid *Cichlidogyrus *were suggested to colonize Cyprinodontiformes as a result of ecological transfer from cichlid hosts [156]. Transfer of marine parasites to cichlids is proposed by Pérez-Ponce de Léon and Choudhury [59] to explain the acquisition of additional parasites (cryptogonimid Digenea) after the cichlid's colonization of Mexico. The authors invoke the salinity tolerance of those cichlids, allowing ecological contact with marine or estuarine fishes and, hence, host-switch events. Another example, for cichlids cultured under marine conditions, is provided by Kaneko et al. [123], reporting the acquisition of the marine capsalid monogeneans *Neobenedenia melleni *(MacCallum, 1927) and *Benedenia monticelli *(Parona and Perugia, 1895) by *Oreochromis mossambicus* (Peters, 1852) and *O. aureus *(Steindachner, 1864), respectively. Those authors mention less-than-optimal adaptation to marine conditions as cause for the susceptibility of the cichlids to a parasite not normally adapted to those hosts, although *N. melleni* has a rather wide host range anyway. 

Hence, freshwater/marine transfers are possible and a marine environment lowering the cichlid's condition could ease the colonization by parasites not specialised in cichlids. In favour of the dispersal hypothesis, one could argue that marine migration, freeing cichlids of other ectoparasitic Monogenea and hence removing interspecific competition, would be an additional facilitating factor for ecological transfer. However, it is often assumed that interspecific competition is not such an issue in Monogenea [157–159]. Freshwater/marine transitions would seem to contradict the above-mentioned limited tolerance to osmotic shocks. However, those ecological transfers across salinity borders should be thought of mostly on an evolutionary timescale, and as a feature occurring in certain genera with a broad salinity tolerance ([100] for *Protogyrodactylus*), rather than as frequent events in a monogenean lifespan.

### 5.3. A Putative Scenario Assuming the Dispersal Hypothesis

The Gondwanan vicariance hypothesis implies isolation of fish populations remaining in their respective freshwater environments. In contrast, in case the present-day distribution pattern of the radiation of cichlids would be the result of dispersal, the hypothesis of Murray [25] applied to their parasites gives us the following scenario. We do not mean to say that the current evidence unequivocally points in this direction; we only outline this scenario as a “thought experiment” as it could explain the present continental differences between cichlid fish parasites. It should also be clear that, whatever the nature of the intercontinental migrations, the present-day cichlid distribution is a result of both vicariance and dispersal. For instance, intracontinental migrations (through freshwater systems) have happened in either case, and have caused the ectoparasites to display a substantial similarity across the respective continents.

1. If Madagascar is indeed the centre of origin of Cichlidae, *Insulacleidus* probably represents the only extant representative of the most basal clade of ectoparasitic Monogenea associated to this fish family. In view of the very simple morphology of its haptoral hard parts, this seems an acceptable assumption.
2. Two marine migrations from this island took place in the late Cretaceous (50–55 MYA [25]), one towards Asia, and another one towards Africa, leading to putative loss of all Malagasy ectoparasites and subsequent infection by parasites of different host families in the new continent. This created the current differences in ectoparasitic fauna between the continents (Figure 1). Mesoparasites, however, were retained during marine migrations. The difference observed for these parasites (*Enterogyrus *spp.) is the result of simple isolation-by-distance. If this were the case, no reproduction would have occurred (or been required) during the migration. Salinity would have posed an obstacle to reinfection during the migration because monogenean larvae (oncomiracidia) are free-living organisms and would not survive this salinity change. The retention of mesoparasites would have been possible, however, if the migrations occurred quickly, allowing the individual adult parasites of migrating hosts to survive the migration. It is important to note that *Enterogyrus*, just like *Urogyrus*, is only known from cichlid hosts [78]. There is, hence, no reason to assume that the presence of representatives of this genus on different continents is a consequence of host-switch from other hosts.
3. A second migration through marine waters took place between West Africa and South America (20 MYA [25]), with the same consequences as above: loss of African ectoparasites, infestation of newly arrived “clean” hosts by South American ectoparasites coming from other host families. The time for migration (from Africa to America) was estimated (based on palaeoreconstructions and with dispersal through the aid of oceanic currents and shallow water areas) at 23 days [25], which is compatible with the survival of mesoparasites without reproduction (*cfr. supra*). This implies that *Enterogyrus*, or a closely related sister clade, might be present in South American cichlids (the only observation of this genus in the Americas is from an introduced African species [117]). Furthermore, even without having to invoke open-oceanic migration, northern landbridges and stretches of less saline seas provided dispersal pathways for freshwater fishes between Europe and the Americas during the late Cretaceous and Tertiary [13]. Thus, the cichlid fossils of Europe ([25]; *cfr. infra*) could fit well in this hypothesis. Also, the still ongoing debate on whether the salinity tolerance of cichlids suffices for oceanic migration would pose no problem here, as dispersal over landbridges or through diluted marine or coastal environments would not require them to withstand marine open-water conditions. In fact, it seems harder to find a plausible dispersal path for cichlids towards Asia. In contrast, for Mastacembelidae, a fish family with an African-Asian distribution where palaeontological and molecular evidence are in favour of dispersal rather than vicariance, a land bridge (over the Middle East) rather than marine migration is suggested as pathway [160]. The Middle East is also known as a centre of exchange for other freshwater fishes, for example, in cyprinids [161].
4. Intracontinental migrations lead to the wider colonization of Africa and South America. As these migrations occurred only in freshwaters, there were no losses of parasites, and the parasite fauna now observed is remarkably homogeneous. Mendoza-Franco and Vidal-Martínez [162] propose an example of this, in *Sciadicleithrum*, which would have migrated with its cichlid hosts from South to Central America after the uplift of the Panama Isthmus. An exception for Africa is *Onchobdella*, only infecting hosts with a distribution limited to West Africa (though its mesoparasitic putative sister *Urogyrus* is more widespread in the continent, *cfr. supra*). In view of Figure 2, their ancestor probably colonized cichlids after lateral transfer from another fish host family.
5. During the African or American intracontinental expansion of the Cichlidae, three lineages isolate themselves: one colonized the Levant (35 MYA [25]), another one Iran, and the last one North America (Rio Grande river, where *Herichthys cyanoguttatus* Baird and Girard, 1854 presently occurs [163]). In her paper, Murray [25] does not conclude whether those fishes migrated through marine or freshwater systems (Figure 3, black and white striped arrows). As Levantine cichlid parasites (ecto- and meso-) are similar to African ones, we suppose that the migrations which introduced this fish family to this area solely involved crossing freshwaters (for otherwise, the ectoparasitic fauna should be different). While Werner and Mokady [164] suggest much more recent colonisation for the only Levantine haplochromine, *Astatotilapia flaviijosephi *(Lortet, 1883), they do not invoke marine dispersal for its arrival from Africa into the Levant either. For the Iranian or North American colonization, we should be able to assess the likelihood of marine or freshwater migration by describing ectoparasites from those cichlid species. Ancyrocephalidae have hitherto not been found on them; only 20 specimens, provided by B. Jalali, of *Iranocichla hormuzensis* have been studied, yielding only gyrodactylid monogeneans (A. Pariselle, unpublished data). No data were available for Rio Grande fishes. In both cases (Iran and North America) *Enterogyrus* (or a related genus—being a mesoparasite) should be present.
6. The presence of cichlid fossils is demonstrated in Europe (Italy) and Central America (Haiti). As these faunas are extinct, their parasites will remain unknown and their migration pathways cannot be inferred from host-parasite data (Figure 3, grey arrows).

## 6. Conclusions and Suggested Approach

The current knowledge on diversity and distribution of parasites does not allow us to be conclusive in supporting either the dispersal or vicariance hypothesis explaining the present-day distribution of cichlids. The interesting outcome of analysing the parasite data is that the resulting conclusions may be tested and falsified. Although the presence of *Enterogyrus* on cichlid hosts worldwide (i.e., in South America and/or Madagascar) has not been demonstrated yet, the most crucial argument here could be provided by a sound phylogeny of dactylogyridean ectoparasites of cichlids (for which the data are currently lacking). In view of classical problems in molecular phylogenetic reconstruction, such as introgression [165] and discordance between conclusions based on mitochondrial *versus* nuclear markers [166], multigene approaches are recommended. One should examine, using molecular phylogenetics, whether there consistently is a closer relationship between Asian, African, or South American cichlid ectoparasites with other ectoparasites from different local host families, than with the parasites of cichlid hosts on other continents. Apart from sistergroup relations, genetics could also give us clues on the evolutionary distances between the various cichlid monogeneans. This is crucial, as the mesoparasite *Enterogyrus* could have a slower rate of evolution and diversification than the various ectoparasitic genera (perhaps due to high constraints resulting from its mesoparasitic lifestyle), possibly explaining the higher diversity in ecto- than in mesoparasites. Though little molecular evolutionary data exist of these animals, results of Mendlová et al. [100] do not support this hypothesis. Moreover, ectoparasitic Monogenea may depend for their speciation on their hosts' diversification ([167] but see [168]), making a speciation burst independent of cichlid history unlikely. Furthermore, mesoparasitism is a derived and polyphyletic feature in our cladogram (and a derived character in the phylogeny of Mendlová et al. [100]), so there is little reason to assume that those parasites in general evolve in a different way from their ectoparasitic counterparts.

Finally, we hope to have exemplified that the evolution and biogeography of parasites should be considered in association with a sound knowledge of their hosts. Indeed, Murray's theory on age and dispersal pattern of cichlids could be in agreement with their continent-specific fauna of ectoparasitic Monogenea, though extra (molecular) data are evidently needed to be conclusive. Conversely, we have to keep in mind the contribution that the study of parasites can make to investigations concerning their hosts, at different levels. This spans from the most specific, where parasites can assist in the identification of host sister species [74, 169], up to the broadest, as in the example presented here, where parasites might lead us to choose between two hypotheses on host origin. On an intermediate level, parasites might resolve an ambiguity on the mode of cichlid biogeographical evolution: for example, that the Levantine migration occurred by crossing freshwater rather than marine systems.

## Figures and Tables

**Table 1:** Genera from non-cichlid hosts included in the phylogenetic analysis, with their host range.

Genus	Host fish families	Reference
*Diplectanum *Diesing, 1858	Muraenesocidae, Gerreidae, Kuhliidae, Latidae, Lutjanidae, Moronidae, Percichthyidae, Polynemidae, Priacanthidae, Sciaenidae, Serranidae, Sillaginidae, Sphyraenidae, Synancejidae, Terapontidae, Toxotidae, Cynoglossidae, Bagridae	[96]
*Mastacembelocleidus *Kritsky, Pandey, Agrawal, and Abdullah, 2004	Mastacembelidae	[97]
*Euryhaliotrema *Kritsky and Boeger, 2002	Sciaenidae, Haemulidae, Sparidae, Lutjanidae	[69]
*Chandacleidus *Agrawal, Tripathi, and Devak, 2006	Freshwater Ambassidae	[98]
*Duplaccessorius *Viozzi and Brugni, 2004	Freshwater Percichthyidae	[99]
*Protogyrodactylus *Johnston and Tiegs, 1922	Marine Terapontidae; Marine and brackish water Gerreidae	[100, 101]
*Quadriacanthus *Paperna, 1961	Clariidae and Bagridae	[102]

## Appendix

(1) Hook: shank tapering proximally; shank with bulb at proximal end. (2) Hooks: similar in shape and size; hook 5 almost splinter-like; greatly variable in size. (3) Thumbs of hooks: erected; straight; depressed. (4) Number of distinct portions in hook shank: one; two. (5) Ventral anchor: fully developed; splinter-like; one fully developed, another splinter-like. (6) Ventral bar—anterior margin: without obvious ornamentation; with subterminal flaps; with large shield-like plate; with small flap. (7) Ventral bar shape: straight; V-shaped; inverted V-shaped; arched. (8) Longitudinal groove on ventral bar: absent; present. (9) Dorsal bar: single; double; absent. (10) Dorsal bar—subterminal anterior flaps: absent; present, well developed and supported by ridges; present, no supporting ridges. (11) Dorsal bar—anterior auricles: absent; present. (12) Dorsal bar: straight or slightly V-shaped; M-shaped; inverted V-shaped. (13) Accessory piece: present; absent. (14) Male copulatory organ: coiled; somewhat straight or straight. (15) Articulation of copulatory complex: articulated; nonarticulated. (16) Vagina: ventral; dextrolateral; sinistrolateral. (17) Vagina: sclerotized; nonsclerotized.
